# Molecular mechanisms and management of traumatic brain injury – missing the link?

**DOI:** 10.1186/1749-7922-4-10

**Published:** 2009-03-04

**Authors:** Michael A Flierl, Wade R Smith, Steven J Morgan, Philip F Stahel

**Affiliations:** 1 Department of Orthopaedic Surgery, Denver Health Medical Center, University of Colorado School of Medicine, 777 Bannock Street, Denver, CO 80204, USA

## 

We read with great interest the recent review article by Veenith *et al*. published in the *World Journal of Emergency Surgery *[1]. In this paper, the authors provide an overview on the epidemiology and pathophysiology of traumatic brain injury (TBI), and present an update on TBI-induced apoptosis, intracranial gene regulation and pharmacological approaches to ameliorate secondary brain injury. The authors are to be congratulated for outlining this important and constantly evolving topic of global importance. Unfortunately, our initial excitement about this paper, which promised to disclose the "missing link" between molecular pathology and new treatment concepts for TBI [1], was not justified. We believe that important pathways in the pathophysiology of TBI and resulting therapeutic concepts were not addressed in the review article. We would therefore like to comment on the missing aspects in the article by Veenith and colleagues [1], in order to provide a more balanced and comprehensive perspective on the topic.

Beyond a doubt, a detailed description of the molecular neuropathology of TBI represents a challenging task, which is difficult to describe in just a few paragraphs. However, the authors could have expanded their article to include some of what we consider "key" pathways in the cellular and molecular pathophysiology of TBI (Figure 1). For example, the role of neurotoxic proteases, nitric oxide and phospholipases released by damaged tissue, the impact on blood-brain-barrier breakdown by recruited and local inflammatory cells, and the activation of the innate immune system, e.g. the complement system, as a crucial mediator of posttraumatic neuroinflammation, are not mentioned or discussed in the paper. The section devoted to apoptosis provides the reader with some basic textbook information and definitions, but may have benefited from an additional update on the current literature in the field of neuronal apoptosis in TBI. Similarly, the paragraph on gene regulation appears to represent a random selection of candidate genes without a rationale being provided on how alterations in gene regulation may relate to the pathophysiology of TBI. Several references cited refer to studies related to cardiovascular disease, rather than head injury. Most importantly, this section of the manuscript fails to stress the clinical relevance of pathological alterations in gene expression.

**Figure 1:**
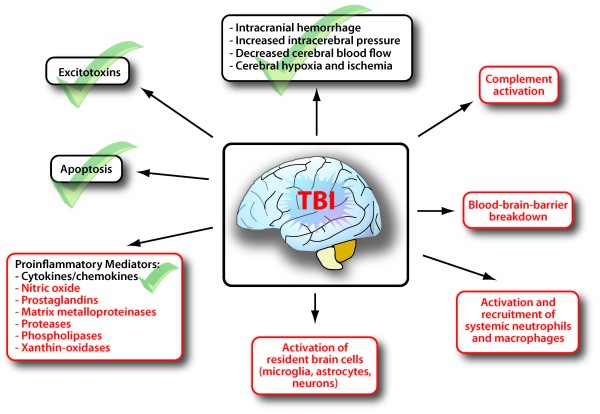
**Simplified schematic of the complex neuroinflammatory response following traumatic brain injury**. Check-marks indicate the areas discussed in the review article by Veenith and colleagues [1], while the red boxes designate pathophysiological pathways of additional interest.

Finally, as a minor comment, the authors should pay more attention to accuracy in the citation of the pertinent literature. For example, reference #10 is claimed to support a statement on interleukins and cerebral edema, when in fact the citation refers to a publication on programmed cell death in nematodes. Several other examples of inadequate reference to the literature could be mentioned. Finally, the title chosen by the authors appears problematic. The authors claim to provide the *"missing link" *between molecular mechanisms and therapeutic concepts in TBI. Unfortunately, the review article fails to provide a bridge between the two entities. In addition, many of the current therapeutic approaches and promising new strategies in search of the pharmacological "golden bullet" are missing [2]. While alterations in gene expression may be an interesting finding and promising target for future scientific approaches, we are still far from bringing the gene therapy concept from "bench to bedside" for an acute traumatic disorder such as TBI.

In summary, we realize that providing an encompassing and scientifically accurate review on the topic represents a virtually impossible task. We are therefore grateful for the review by Veenith *et al*. [1] and we hope to contribute to the authors' search of the "missing link" between molecular pathophysiology and new therapeutic concepts in TBI by the identification of additional pathways of interest (Fig. 1).

## Competing interests

The authors declare that they have no competing interests.

## Authors' contributions

MAF and PFS wrote the manuscript. WRS and SJM critically revised the paper. All authors approved the final version of this manuscript.

## References

[B1] Veenith T, Goon SH, Burnstein RM (2009). Molecular mechanisms of traumatic brain injury – the missing link in management. World J Emerg Surg.

[B2] Beauchamp K, Mutlak H, Smith WR, Shohami E, Stahel PF (2008). Pharmacology of traumatic brain injury: where is the "golden bullet"?. Mol Med.

